# Improving Physicians’ Knowledge of Emergency Oxygen Therapy According to British Thoracic Society (BTS) Guidelines: A Closed-Loop Clinical Audit at Al-Managil Teaching Hospital

**DOI:** 10.7759/cureus.117028

**Published:** 2026-09-27

**Authors:** Muram Mohamed Elmahadi Ali Salih, Ahmed G. N Suliman, Elkhatab Abdulrahman Mustafa Abdulrahman, Mohammed Omer Elbadawi Elhassan, Abdulrahman Abbas Yusuf Mohammed, Wassan Hassan Mohammed Elmowag, Hala Aamir Abdelraouf Ahmed, Duaa Hussain Mahmoud Mohamed, Ibrahim Abdelhafiz Ibrahim Ahmed, Amal Muawia Hussein Elamen, Salaheldin Mohamed Musa Hassan, Elsheikh Elamin Ahmed Elamin, Abdalmahmoud Asadig Kanan Ahmed, Abdelgalil Esameldin Omer Eldaw, Zainab Maqboul Hasan Mohamed, Dina Abdelnaser Ahmed Hussein, Aya Abdelfattah Kaml Hassan, Israa Tariq Balla Abdalla, Amal Egbal Ibrahim Eisa, Aisha Babiker Sobahy Mohamed, Mustafa Mohamed

**Affiliations:** 1 Medicine, National Ribat University, Khartoum, SDN; 2 Internal Medicine, Al-Managil Teaching Hospital, Al-Managil, SDN; 3 Internal Medicine, University of Medical Sciences and Technology, Khartoum, SDN; 4 Emergency and Acute Medicine, Response Plus Medical Services, Abu Dhabi, ARE; 5 Internal Medicine, Hamad Medical Corporation, Doha, QAT; 6 Internal Medicine, Port Sudan Teaching Hospital, Port Sudan, SDN; 7 Internal Medicine, University of Gezira, Wad Madani, SDN; 8 Medicine, Al-Managil Teaching Hospital, Al-Managil, SDN; 9 Internal Medicine, Dongola Specialized Hospital, Dongola, SDN; 10 Medicine, Al-Managil Teaching Hospital, Managil, SDN; 11 Surgery, Almanagil Teaching Hospital, Almanagil, SDN; 12 Emergency, Al-Managil Teaching Hospital, Al-Managil, SDN; 13 Internal Medicine and General Surgery, Al-Managil Teaching Hospital, Al-Managil, SDN; 14 Emergency Medicine, Al-Managil Teaching Hospital, Al-Managil, SDN; 15 Emergency Department, Al-Managil Teaching Hospital, Al-Managil, SDN; 16 General Physician, Assiut University Hospital, Assiut, EGY; 17 Emergency Medicine, University of Sharjah, Sharjah, ARE; 18 Internal Medicine, Assiut University Hospital, Assiut, EGY; 19 Internal Medicine, AL-Neelain University, Khartoum, SDN

**Keywords:** british thoracic society, clinical audit, emergency oxygen, internal medicine, medical education, oxygen prescribing, oxygen therapy, quality improvement

## Abstract

Background

Oxygen is an essential medication that should be prescribed and administered according to evidence-based guidelines to prevent both hypoxaemia and hyperoxia. Despite the availability of the British Thoracic Society (BTS) guideline, deficiencies in physicians' knowledge of oxygen therapy remain common and may compromise patient safety. This study aimed to assess changes in physicians' knowledge of evidence-based oxygen therapy following a structured educational intervention through a closed-loop clinical audit.

Methods

A prospective closed-loop clinical audit was conducted in the Internal Medicine Department of Al-Managil Teaching Hospital, Sudan. A total-coverage sampling strategy was employed, with 64 physicians participating in the baseline audit and 64 physicians participating in the re-audit. Knowledge was assessed using the same structured 15-item questionnaire derived from BTS oxygen therapy standards. Following the baseline assessment, a one-month multifaceted educational intervention was implemented. The second audit cycle was conducted using the same assessment tool, and physicians' knowledge before and after the intervention was compared.

Results

Knowledge improved across all fifteen assessed BTS standards following the educational intervention. The greatest improvement was observed in prescribing target oxygen saturation ranges for all admitted patients, which increased from 16 (25.0%) to 37 (57.8%) (χ² = 14.20, p < 0.001). Significant improvements were also observed in knowledge of oxygen humidification, pulse oximetry limitations, arterial blood gas interpretation, oxygen prescription documentation, and oxygen-induced hypercapnia. Overall, improvements ranged from 14.1 to 32.8 percentage points across the assessed standards. Despite these gains, only one standard exceeded the predefined performance target, indicating persistent knowledge gaps requiring further quality improvement.

Conclusion

A structured educational intervention was associated with significant improvements in departmental-level physicians' knowledge of evidence-based oxygen therapy. However, persistent deficiencies in several key domains highlight the need for continuous professional education, regular re-audit, and ongoing quality improvement initiatives to strengthen guideline adherence and promote safer oxygen prescribing practices.

## Introduction

Oxygen is one of the most frequently administered treatments in acute medical care and is considered an essential medicine for the management of hypoxaemia. However, unlike many other medications, oxygen has historically been administered empirically, often without a formal prescription or clearly defined therapeutic target. Contemporary evidence recognises oxygen as a drug that should be prescribed, administered, monitored, and titrated according to individual patient requirements. Both inadequate oxygenation and excessive oxygen administration may result in adverse clinical outcomes, highlighting the importance of evidence-based oxygen therapy [1-3].

The primary indication for supplemental oxygen is the correction of hypoxaemia rather than the treatment of breathlessness alone. The British Thoracic Society (BTS) guideline recommends target oxygen saturation ranges of 94-98% for most acutely ill adults and 88-92% for patients at risk of hypercapnic respiratory failure, including many individuals with chronic obstructive pulmonary disease (COPD). Individualised target saturation ranges, appropriate oxygen delivery devices, continuous pulse oximetry, and accurate documentation of oxygen prescriptions are fundamental components of safe oxygen therapy [1].

Increasing evidence indicates that excessive oxygen administration is not benign. Liberal oxygen therapy has been associated with increased mortality and poorer clinical outcomes in acutely ill adults compared with conservative, target-based oxygen strategies. Hyperoxia may contribute to pulmonary toxicity, oxidative stress, vasoconstriction, and worsening hypercapnia in susceptible patients, whereas failure to recognise and appropriately treat hypoxaemia can result in tissue hypoxia and organ dysfunction. These findings have reinforced international recommendations advocating judicious, target-driven oxygen administration rather than routine high-concentration oxygen therapy [1,3].

Despite the availability of comprehensive evidence-based guidelines, studies have consistently demonstrated deficiencies in clinicians' knowledge and adherence to recommended oxygen prescribing practices. Common problems include inappropriate target oxygen saturation selection, inadequate understanding of oxygen delivery devices, poor documentation of oxygen prescriptions, and limited awareness of the risks associated with both hypoxaemia and hyperoxia. These deficiencies may contribute to inappropriate oxygen administration and compromise patient safety [1,4-6].

Clinical audit is a recognised quality improvement methodology that systematically compares current practice against explicit evidence-based standards, implements targeted interventions to address identified deficiencies, and subsequently reassesses performance to determine whether meaningful improvement has been achieved. Closed-loop clinical audits are particularly valuable because they evaluate the effectiveness of implemented changes and promote sustainable improvements in clinical practice through repeated measurement and feedback [1].

Therefore, this closed-loop clinical audit was conducted in the internal medicine department of Al-Managil Teaching Hospital using the BTS guideline as the audit standard. The objectives were to reassess physicians' knowledge of emergency oxygen therapy following a structured educational intervention, compare post-intervention performance with baseline findings across fifteen predefined BTS knowledge standards, identify persistent knowledge gaps requiring further quality improvement, and assess changes in departmental-level physicians' knowledge following the educational programme.

## Materials and methods

Study design

A prospective, closed-loop clinical audit was conducted to assess and improve physicians’ knowledge of evidence-based oxygen therapy. The audit followed the conventional clinical audit cycle: identification of evidence-based standards, baseline measurement, implementation of a targeted educational intervention, and re-audit using the same standards and assessment instrument. The project was reported with reference to the principles of the Standards for Quality Improvement Reporting Excellence (SQUIRE 2.0) [7].

Study setting

The audit was conducted in the internal medicine department of Al-Managil Teaching Hospital, Sudan. The department provides inpatient medical services and is staffed by physicians responsible for the assessment, prescribing, monitoring, and management of oxygen therapy in adult patients.

The baseline audit was conducted over one month between 16 March and 15 April 2026. This was followed by a one-month educational intervention implemented from 16 April to 15 May 2026. The second audit cycle was subsequently undertaken over approximately two months between 16 May and 10 July 2026 to evaluate the impact of the intervention.

Study population

The target population comprised physicians working in the internal medicine department during the respective audit periods. Eligible participants included house officers, medical officers, registrars, specialists, and other physicians involved in the assessment or management of adult medical patients.

Physicians were eligible if they were working in the internal medicine department during the relevant audit cycle; were involved in prescribing, administering, monitoring, or reviewing oxygen therapy; and voluntarily agreed to participate.

Physicians who were absent throughout the data-collection period, were not involved in adult patient care, or submitted substantially incomplete questionnaires were excluded from the analysis.

Sampling technique and sample size

A total-coverage sampling technique was used in both audit cycles. All physicians who met the eligibility criteria and were available in the Internal Medicine Department during each data-collection period were invited to participate.

A total of 64 physicians completed the baseline assessment, and 64 physicians completed the second-cycle assessment. Participation in the two cycles was analysed at the group level. Because questionnaires were completed anonymously and no unique participant identifiers were collected, individual responses could not be paired across the two cycles. The two datasets were therefore treated as independent departmental samples unless subsequent project records confirm that identifiable paired data were available.

Audit criteria and standards

The audit criteria were derived primarily from the BTS guideline for oxygen use in adults in healthcare and emergency settings. Fifteen predefined knowledge standards were selected to assess key aspects of safe oxygen prescribing, administration, monitoring, and documentation.

The assessed standards covered the following domains: recommended target oxygen saturation of 94-98% for most acutely ill adults; recommended target saturation of 88-92% for patients at risk of hypercapnic respiratory failure; recognition of type 1 respiratory failure; mechanisms contributing to oxygen-induced hypercapnia; risks associated with sudden withdrawal of supplemental oxygen; limitations of earlobe capillary blood-gas measurements; limitations of pulse oximetry in carbon monoxide poisoning; minimum oxygen flow required when using a simple face mask; indications for oxygen humidification in patients with an artificial airway; potential overestimation of oxygen saturation by pulse oximetry in individuals with darker skin pigmentation; prescription of oxygen according to a defined target saturation range; documentation of a target saturation range for admitted patients; completion of the prescriber’s signature, printed name, and date; use of an oxygen alert card while awaiting blood-gas results; and completion of all essential components of an oxygen prescription [1].

Each standard was assessed through one corresponding multiple-choice question. A correct response was awarded one point, while an incorrect or unanswered response received zero points. The maximum possible individual knowledge score was therefore 15.

For the purpose of interpreting departmental performance, 80% was used as the desired level of achievement for each knowledge standard. The use of an 80% threshold should be described as a locally selected quality-improvement target rather than a formal BTS compliance threshold unless the guideline explicitly specifies that level.

Audit instrument

Data were collected using a structured, self-administered electronic questionnaire developed by the audit team specifically for this clinical audit based on recommendations from the BTS guideline for oxygen use in adults in healthcare and emergency settings [1]. The questionnaire consisted of 15 multiple-choice questions mapped to the predefined audit standards and assessed physicians' knowledge of oxygen target ranges, respiratory physiology, oxygen delivery devices, pulse oximetry limitations, blood-gas interpretation, prescription requirements, and oxygen-related safety precautions. The complete questionnaire is provided in Appendix 1.

The same questionnaire, question sequence, scoring method, and audit standards were used in both cycles to preserve comparability between baseline and post-intervention findings.

Before data collection, the questionnaire was reviewed independently by three senior Internal Medicine consultants with experience in respiratory medicine, clinical audit, and evidence-based practice to assess its clinical relevance, clarity, and consistency with BTS guideline recommendations. Content validity was supported through this expert review and the direct mapping of each questionnaire item to a predefined BTS-based audit standard. Minor modifications were made to improve the wording and clarity of several items before final distribution. As the questionnaire was developed specifically for this clinical audit based on established BTS recommendations, no formal psychometric validation or pilot testing was undertaken. Formal test-retest reliability assessment was not performed; however, measurement consistency between cycles was supported by using the same questionnaire, question sequence, scoring method, and administration procedures.

Baseline audit cycle

During the first audit cycle, all eligible physicians were invited to complete the electronic questionnaire. Participants received a brief explanation of the project purpose and were informed that participation was voluntary and anonymous. To minimise consultation between participants and reduce contamination of responses, the questionnaire was completed individually under similar instructions.

No educational material containing the questionnaire answers was provided immediately before completion of the baseline assessment. Responses were automatically recorded through Google Forms and subsequently exported into a spreadsheet for data cleaning and analysis.

Baseline results were calculated for each of the 15 standards. The findings were reviewed by the audit team to identify areas of adequate knowledge and domains requiring targeted improvement. Particular deficiencies were identified in prescribing a target saturation range for all admitted patients, oxygen humidification, limitations of pulse oximetry in darker skin pigmentation, interpretation of earlobe capillary blood gases, and completion of oxygen-prescription documentation.

Educational intervention

Following analysis of the baseline findings, a structured, multifaceted educational intervention was implemented over one month (16 April-15 May 2026). The intervention was designed to address the knowledge deficiencies identified during the first audit cycle and was based on the recommendations of the BTS guideline for oxygen use in adults [1].

The educational programme included interactive teaching sessions on oxygen as a prescribed medication, review of recommended target oxygen saturation ranges, case-based discussions on common clinical scenarios, instruction on appropriate oxygen delivery devices and flow rates, education on oxygen-induced hypercapnia and rebound hypoxaemia, interpretation of pulse oximetry and blood gas measurements, guidance on oxygen prescription and documentation, dissemination of educational materials, and feedback on the deficiencies identified during the baseline audit. Participants were also given opportunities to discuss common misconceptions and ask questions during the educational sessions.

The intervention was delivered by the clinical audit team using standardized guideline-based educational materials to ensure consistency throughout the programme. Attendance at the educational sessions and individual receipt of the educational materials were not formally recorded. Consequently, individual-level exposure to the intervention among physicians participating in the re-audit could not be reliably established.

Second audit cycle

Following completion of the intervention, the audit was repeated using the same eligibility criteria, total-coverage sampling approach, 15-item questionnaire, data-collection method, and scoring system applied during the baseline cycle.

All eligible physicians working in the department during the second-cycle period were invited to participate. A total of 64 completed questionnaires were included in the re-audit. The second-cycle results were compared with baseline findings to evaluate the effect of the educational intervention and determine whether previously identified knowledge gaps had improved.

Because participation was anonymous and individual respondents could not be reliably linked between cycles, the analysis evaluated change at the departmental level rather than individual learning trajectories.

Outcome measures

The primary outcome was the proportion of correct responses for each of the 15 audit standards in the second cycle compared with the corresponding proportion in the baseline cycle.

Secondary outcomes included the absolute percentage-point change for each standard, the number of standards demonstrating improvement, the overall mean or median knowledge score per participant if individual total scores were available, the proportion of participants achieving a predefined satisfactory overall score, and the identification of standards that remained below the locally defined performance target after the intervention. Absolute improvement was calculated by subtracting the baseline correct-response percentage from the corresponding second-cycle percentage.

Data management and statistical analysis

Data were exported from Google Forms (Google LLC, Mountain View, USA) into Microsoft Excel (Microsoft Corporation, Redmond, WA, USA) for cleaning and coding before being analysed using JASP version 0.96.0 (JASP Team, University of Amsterdam, Amsterdam, The Netherlands).

Categorical variables were summarised as frequencies and percentages. For each of the 15 audit standards, the number and proportion of physicians providing the correct response were calculated for both the baseline audit and the re-audit. Differences in the proportions of correct responses between the two audit cycles were compared using Pearson's chi-square test. Fisher's exact test was considered when the assumptions of the chi-square test were not met.

The magnitude of improvement was expressed as the absolute percentage-point change between the baseline audit and the re-audit for each standard. Statistical significance was determined using two-sided tests, and a p-value < 0.05 was considered statistically significant.

Measures to promote data quality

Several measures were implemented to ensure data quality and consistency throughout both audit cycles. The same questionnaire, scoring criteria, and data-collection procedures were used in both cycles. Questionnaire items were mapped directly to the predefined BTS audit standards, and standardized instructions were provided to all participants before questionnaire completion [1]. Data were collected electronically using Google Forms, minimizing transcription errors and facilitating accurate data capture. Exported datasets were reviewed for duplicate submissions, completeness, and internal consistency prior to analysis. No duplicate responses were identified, and all submitted questionnaires were complete; therefore, no questionnaires were excluded from the analysis, and no imputation of missing data was required.

Ethical considerations

The audit was conducted as part of the hospital's clinical governance and quality improvement programme following approval by the Institutional Review Board of Al-Managil Teaching Hospital under IRB Reference Number: 24-OT2026.

Participation was voluntary, and electronic informed consent was obtained from all participants before questionnaire completion. Questionnaires were completed anonymously, and no personally identifiable information was collected. All data were stored securely and used exclusively for clinical audit, quality improvement, and scientific reporting purposes. The project complied with internationally recognised principles of clinical governance and institutional ethical standards.

## Results

A total of 64 physicians participated in the baseline audit, and 64 physicians participated in the second audit cycle following implementation of the educational intervention. All completed questionnaires were included in the final analysis.

Overall, physicians' knowledge of evidence-based oxygen therapy improved across all fifteen predefined BTS audit standards following the educational intervention (Table 1). Absolute improvements in correct responses ranged from 14.1% to 32.8%, demonstrating consistent gains across the domains of oxygen physiology, monitoring and delivery, and prescribing and safety.

**Table 1:** Comparison of physicians' knowledge of BTS oxygen therapy standards before and after the educational intervention BTS: British Thoracic Society; PaO₂: arterial partial pressure of oxygen; V/Q: ventilation-perfusion.

Audit standard	Baseline audit n (%) (n=64)	Re-audit n (%) (n=64)	χ²	p-value	Absolute improvement (percentage points)
Targets and physiology					
Target oxygen saturation 94–98% for acutely ill adults	39 (60.9)	50 (78.1)	4.46	0.035	+17.2
Target oxygen saturation 88–92% for patients at risk of hypercapnic respiratory failure	43 (67.2)	52 (81.3)	3.31	0.069	+14.1
Type 1 respiratory failure (PaO₂ <8 kPa)	37 (57.8)	49 (76.6)	5.10	0.024	+18.8
Mechanism of oxygen-induced hypercapnia (V/Q mismatch)	38 (59.4)	49 (76.6)	4.34	0.037	+17.2
Rebound hypoxaemia following abrupt oxygen withdrawal	39 (60.9)	50 (78.1)	4.46	0.035	+17.2
Monitoring and delivery					
Earlobe capillary blood gas underestimates arterial PaO₂	30 (46.9)	45 (70.3)	7.25	0.007	+23.4
Pulse oximetry is unreliable in carbon monoxide poisoning	40 (62.5)	50 (78.1)	3.74	0.053	+15.6
Simple face mask requires a minimum oxygen flow of 5 L/min	40 (62.5)	50 (78.1)	3.74	0.053	+15.6
Humidification is indicated for patients with an artificial airway	29 (45.3)	44 (68.8)	7.17	0.007	+23.4
Pulse oximetry may overestimate oxygen saturation in individuals with darker skin pigmentation	29 (45.3)	44 (68.8)	7.17	0.007	+23.4
Prescribing and safety					
Oxygen should be prescribed according to a target saturation range	35 (54.7)	48 (75.0)	5.79	0.016	+20.3
A target oxygen saturation range should be prescribed for all admitted patients	16 (25.0)	37 (57.8)	14.20	<0.001	+32.8
Oxygen prescription should include the prescriber's signature, printed name, and date	31 (48.4)	45 (70.3)	6.35	0.012	+21.9
Oxygen alert card should be followed until arterial blood gas results are available	31 (48.4)	45 (70.3)	6.35	0.012	+21.9
Complete oxygen prescription (target range, signature, name, and date)	40 (62.5)	50 (78.1)	3.74	0.053	+15.6

Among the targets and physiology standards, knowledge of the recommended target oxygen saturation for acutely ill adults increased from 39 (60.9%) to 50 (78.1%) (χ² = 4.46, p = 0.035). Correct identification of the target saturation range for patients at risk of hypercapnic respiratory failure improved from 43 (67.2%) to 52 (81.3%), although this difference did not reach statistical significance (χ² = 3.31, p = 0.069). Significant improvements were also observed in knowledge of type 1 respiratory failure, oxygen-induced hypercapnia, and rebound hypoxaemia following oxygen withdrawal (Table 1).

Substantial improvements were observed in the monitoring and delivery domain. Knowledge that earlobe capillary blood gases underestimate arterial oxygen tension increased from 30 (46.9%) to 45 (70.3%) (χ² = 7.25, p = 0.007). Similarly, correct responses regarding humidification of patients with an artificial airway and recognition of pulse oximetry overestimation in individuals with darker skin pigmentation increased from 29 (45.3%) to 44 (68.8%) for both standards (χ² = 7.17, p = 0.007). Improvements in knowledge of pulse oximetry limitations in carbon monoxide poisoning and the minimum oxygen flow required for a simple face mask were also observed, although these changes did not achieve statistical significance (Table 1).

The greatest improvements occurred within the prescribing and safety domain. Knowledge that all admitted patients should have a prescribed target oxygen saturation range increased from 16 (25.0%) to 37 (57.8%), representing the largest absolute improvement (+32.8%) and the strongest statistical evidence of change (χ² = 14.20, p < 0.001). Significant improvements were also observed in knowledge of oxygen prescription documentation requirements, including completion of the prescriber's signature, printed name, and date, as well as appropriate use of oxygen alert cards (both χ² = 6.35, p = 0.012). Knowledge of prescribing oxygen according to an individualized target saturation range also improved significantly from 35 (54.7%) to 48 (75.0%) (χ² = 5.79, p = 0.016).

Despite these improvements, several knowledge gaps persisted following the intervention. Only one of the fifteen assessed standards achieved a correct response rate above 80% during the second audit cycle, while the remaining standards continued to fall below the predetermined performance target. The lowest post-intervention performance was observed for prescribing a target oxygen saturation range for all admitted patients (57.8%), followed by humidification for patients with an artificial airway (68.8%), recognition of pulse oximetry overestimation in individuals with darker skin pigmentation (68.8%), interpretation of earlobe capillary blood gas measurements (70.3%), oxygen prescription documentation (70.3%), and use of oxygen alert cards (70.3%), indicating areas requiring further educational reinforcement.

## Discussion

This closed-loop clinical audit demonstrated that a structured educational intervention based on the BTS guideline was associated with consistent improvements in physicians' knowledge across all fifteen predefined oxygen therapy standards [1]. Improvements were observed across oxygen physiology, monitoring and delivery, and prescribing and safety, suggesting that targeted education can address multiple deficiencies in clinicians' understanding of evidence-based oxygen therapy. Nevertheless, although all assessed standards improved, only one exceeded the predefined performance target after the intervention. This residual gap is important because it indicates that a single educational programme, while beneficial, may be insufficient to achieve uniformly high levels of knowledge and reinforces the need for repeated education, audit, feedback, and system-level support.

The greatest improvement in the present audit was observed in physicians' knowledge that a target oxygen saturation range should be prescribed for all admitted patients. This finding is clinically important because oxygen should be treated as a prescribed therapy with a defined therapeutic target rather than administered routinely without titration. The clinical consequences of inappropriate oxygen use extend beyond documentation quality. In the Improving Oxygen Therapy in Acute-Illness (IOTA) systematic review and meta-analysis of 25 randomized controlled trials involving 16,037 acutely ill adults, Chu et al. found that liberal oxygen therapy was associated with increased in-hospital and 30-day mortality compared with conservative oxygen strategies, without improvement in other patient-important outcomes [8]. These findings reinforce the clinical relevance of ensuring that physicians understand target-based oxygen prescribing and avoid unnecessary hyperoxaemia.

Similar improvements in oxygen prescribing have been reported following multidimensional quality improvement interventions. Rose et al., in a project conducted at Hamad General Hospital in Qatar, used repeated plan-do-study-act (PDSA) cycles incorporating educational sessions, electronic prescribing, posters and reminders, nurse-led reinforcement, and repeated teaching. At baseline, no patients had a valid electronic oxygen prescription; following the intervention, electronic oxygen prescribing reached 93%, while documentation of oxygen indication, target saturation, and a weaning plan improved to 85%, 86%, and 80%, respectively [4]. The magnitude of improvement in that study was greater than the knowledge gains observed in our audit, but the interventions were also broader and directly embedded into clinical workflow. This difference suggests that combining education with prescribing-system modifications and multidisciplinary reinforcement may produce greater changes in practice than education alone.

Evidence from Sudan provides an especially relevant comparison. Muhammed et al. conducted a clinical audit of acute oxygen therapy at Atbara Teaching Hospital using BTS-based standards, staff education, and standardized protocols [9]. Following the intervention, documentation of oxygen saturation and target saturation improved to 100%, while compliance with several oxygen delivery measures also increased substantially. However, some aspects remained suboptimal, including specification of the target range for patients with type 2 respiratory failure and timely oxygen discontinuation. Although that audit assessed actual clinical practice whereas the present study assessed physicians' knowledge, both studies demonstrate that structured guideline-based interventions can produce substantial improvement in Sudanese teaching-hospital settings while also revealing residual deficiencies that require further audit cycles. The consistency of these findings across two Sudanese institutions strengthens the rationale for incorporating oxygen-related education and audit into routine hospital quality-improvement programmes.

Comparable observations have been reported by Wangdi et al. in an emergency department quality improvement initiative at the national referral hospital in Bhutan. Through consecutive PDSA cycles involving staff education, regular reminders, improved communication, and optimization of oxygen delivery systems, substantial improvements were reported in appropriate oxygen prescribing and delivery [10]. Their findings support the principle that clinicians' knowledge is an important component of safer oxygen therapy but that sustained improvement is more likely when education is combined with repeated measurement, reinforcement, and changes to the clinical environment.

The importance of combining education with practical reinforcement is also supported by other quality improvement studies. Medford et al. evaluated nurse education together with a nurse-facilitated reminder system in a medical admissions unit and reported an increase in oxygen prescribing from 0% before intervention to 49% afterward [11]. Similarly, earlier audit work examining education on a respiratory ward found that oxygen prescription increased from only 5% before education to 20% afterward, despite substantial improvements in oxygen-delivery problems [12]. Together, these findings suggest that education can improve oxygen-related practice but may have a limited effect when used in isolation. Reminder systems, multidisciplinary participation, standardized prescription processes, and repeated feedback may therefore be necessary to translate improved knowledge into consistent bedside behaviour.

Our findings are also consistent with those of Helliar et al., who reported that tailoring educational interventions to specific healthcare professional groups improved oxygen prescribing within a cardiology setting [13]. Their quality improvement work highlighted the multifactorial nature of deficiencies in oxygen prescribing and the importance of addressing both prescribers and staff responsible for oxygen administration. Similarly, the present audit demonstrated substantial improvement in prescribing-related knowledge, including knowledge of individualized target saturation ranges and prescription documentation. These observations suggest that interventions directed specifically toward identified deficiencies may be more effective than generic education and that future cycles should target the standards that remained weakest after the present intervention.

An additional important finding was the improvement in knowledge regarding oxygen monitoring, including limitations of pulse oximetry in individuals with darker skin pigmentation. This issue has acquired increasing clinical importance because pulse oximeters may overestimate arterial oxygen saturation in individuals with darker skin tones, potentially resulting in occult hypoxaemia and delayed recognition of clinically significant hypoxaemia [14]. Awareness of this limitation is particularly relevant in the population served by our institution. Improvement in this item following education therefore represents more than acquisition of theoretical knowledge; it may improve clinicians' interpretation of apparently reassuring oxygen saturation measurements and encourage consideration of the overall clinical picture and, when appropriate, confirmatory blood-gas assessment.

The improvement in knowledge of humidification, blood-gas interpretation, oxygen-induced hypercapnia, and oxygen prescription documentation further illustrates the breadth of knowledge required for safe oxygen therapy. These domains encompass not only selecting an appropriate oxygen target but also understanding how oxygen is delivered, monitored, adjusted, and documented. The prospective Oxygen Project by Nguyen et al. similarly demonstrated that an educational programme targeting nursing and junior medical staff could improve aspects of oxygen prescribing, although improvements were not uniform or consistently sustained at three and six months [15]. This finding is particularly relevant to the present audit because our re-assessment was conducted relatively soon after the intervention. Whether the observed improvements would persist over a longer period remains uncertain and should be evaluated in future audit cycles.

Despite these encouraging findings, several standards remained below the predefined performance target after the intervention. This pattern is consistent with the broader quality improvement literature, in which improvement in oxygen prescribing is generally greatest when education is accompanied by interventions embedded within routine clinical workflows. Strategies including dedicated oxygen prescription sections, electronic prescribing prompts, nurse-facilitated reminders, point-of-care educational materials, standardized documentation tools, and repeated PDSA cycles have all been used to reinforce appropriate oxygen prescribing [4, 11]. These approaches reduce reliance on clinicians remembering guideline recommendations independently and may therefore help bridge the gap between knowledge acquisition and consistent clinical practice.

The present findings should also be interpreted in light of the distinction between knowledge and behaviour. Our questionnaire measured whether physicians knew the correct BTS-based recommendations; it did not determine whether those recommendations were consistently implemented at the bedside. Previous quality improvement studies have demonstrated that prescribing and administration are influenced not only by knowledge but also by documentation systems, availability of appropriate delivery devices, nursing practice, workflow, reminders, and institutional culture. Consequently, improved questionnaire performance should be regarded as an intermediate outcome rather than evidence of improved patient care itself. A logical next audit cycle would therefore complement knowledge assessment with direct evaluation of oxygen prescriptions, documentation of target saturation ranges, actual oxygen administration, and achievement of prescribed saturation targets.

The present study has several limitations. First, it was conducted in a single teaching hospital, which may limit the generalisability of the findings to other healthcare settings. Second, the audit evaluated physicians' knowledge using a structured questionnaire rather than directly measuring oxygen prescribing behaviour or patient outcomes; therefore, improvements in knowledge cannot be assumed to translate directly into improvements in clinical practice. The questionnaire was developed specifically for this audit and, although it underwent expert review and was mapped to BTS-based standards, it was not formally psychometrically validated, pilot tested, or assessed for test-retest reliability, which may limit the reliability and validity of the knowledge measurements. Third, the post-intervention assessment was performed relatively soon after completion of the educational programme, and long-term retention of knowledge was not evaluated. Fourth, attendance at the educational sessions and individual receipt of the educational materials were not formally tracked; therefore, it could not be established whether all physicians participating in the re-audit had been exposed to the intervention, and variation in exposure may have influenced the observed changes in knowledge. Furthermore, because individual responses could not be reliably paired between audit cycles, changes were assessed at the departmental level rather than as individual learning trajectories. Finally, no adjustment for multiple comparisons was applied across the 15 audit standards, and statistically significant findings with p-values close to 0.05 should therefore be interpreted with caution because of the potential for type I error.

Despite these limitations, the audit has several important strengths. It employed a prospective closed-loop clinical audit design, used a total-coverage sampling strategy, applied predefined evidence-based BTS standards, and evaluated the intervention using the same assessment instrument in both audit cycles. The addition of evidence from other international quality improvement projects and, importantly, from another Sudanese teaching hospital places the findings within a broader context. Collectively, this evidence suggests that structured education can improve clinicians' understanding of safe oxygen therapy, but sustained improvement is likely to require repeated audit cycles and integration of educational, multidisciplinary, and system-level interventions into routine clinical practice.

## Conclusions

This closed-loop clinical audit demonstrated that a structured educational intervention was associated with significant improvements in departmental-level physicians' knowledge of evidence-based oxygen therapy across all assessed BTS standards. Given the unpaired audit cycles and the inability to establish individual exposure to the intervention, these findings represent departmental-level changes and should not be interpreted as definitive evidence of individual learning or a causal intervention effect. However, several knowledge gaps persisted after the intervention, indicating the need for ongoing education, regular re-audit, and continuous quality improvement initiatives. Sustained educational reinforcement and adherence to guideline-based oxygen prescribing may contribute to safer clinical practice and improved patient care.

## Appendices

Appendix 1 

**Table 2:** Questionnaire used to assess physicians' knowledge of BTS oxygen therapy recommendations The 15-item multiple-choice questionnaire was developed by the audit team specifically for this clinical audit, based on recommendations from the BTS guideline for oxygen use in adults in healthcare and emergency settings. Each item had one correct response. Correct responses were awarded one point, while incorrect responses were awarded zero points, giving a maximum total score of 15. BTS: British Thoracic Society; COPD: chronic obstructive pulmonary disease; PaO₂: arterial partial pressure of oxygen; PaCO₂: arterial partial pressure of carbon dioxide; HCO₃⁻: bicarbonate; V/Q: ventilation-perfusion; FiO₂: fraction of inspired oxygen; CO₂: carbon dioxide.

No.	Questionnaire item	Response options
1	Recommended target oxygen saturation range for acutely ill patients NOT at risk of hypercapnic respiratory failure?	A. 88–92% • B. 90–94% • C. 94–98% • D. 98–100%
2	Initial target oxygen saturation range for patients with known COPD or other risk factors for hypercapnic respiratory failure?	A. 85–89% • B. 88–92% • C. 92–96% • D. 94–98%
3	How is Type 1 respiratory failure defined in terms of PaO₂?	A. PaO₂ < 6.0 kPa • B. PaO₂ < 8.0 kPa • C. PaO₂ > 10.0 kPa • D. PaO₂ > 13.0 kPa
4	Primary physiological mechanism for oxygen-induced hypercapnia in COPD?	A. Complete loss of hypoxic respiratory drive • B. Worsening of ventilation–perfusion (V/Q) matching • C. Widespread absorption atelectasis • D. Decreased blood viscosity
5	Which parameter is less accurately measured by an arterialised earlobe capillary blood gas compared with a direct arterial sample?	A. pH • B. PaO₂ • C. PaCO₂ • D. HCO₃⁻
6	Why is standard pulse oximetry unreliable in carbon monoxide poisoning?	A. It underestimates true arterial saturation • B. It cannot detect pulsatile flow • C. It cannot differentiate carboxyhaemoglobin from oxyhaemoglobin • D. It is disrupted by tachycardia
7	Minimum recommended flow rate for a simple face mask to prevent CO₂ rebreathing?	A. 2 L/min • B. 5 L/min • C. 10 L/min • D. 15 L/min
8	When is humidification of supplemental oxygen routinely recommended in the emergency setting?	A. For all patients on emergency oxygen • B. For nasal cannulae at 2 L/min • C. Confined to patients with a tracheostomy or artificial airway • D. Contraindicated in acute respiratory failure
9	Major risk if high-concentration oxygen is suddenly withdrawn from a patient with oxygen-induced hypercapnia?	A. Rebound hypoxaemia • B. Severe bronchospasm • C. Instantaneous hypocapnia • D. Tension pneumothorax
10	Known limitation of pulse oximetry in patients with dark skin pigmentation?	A. Underestimates saturation at all levels • B. Overestimates saturation at levels below 80–85% • C. Cannot measure heart rate during hypoxaemia • D. Detects methaemoglobin but not carboxyhaemoglobin
11	Oxygen should be prescribed as:	A. A fixed concentration (%) • B. A fixed FiO₂ • C. A target oxygen saturation range • D. A fixed flow rate in L/min
12	The guideline states a target saturation range should be prescribed for:	A. Only critical care patients • B. Only patients at risk of hypercapnic respiratory failure • C. All patients admitted to hospital • D. Only patients with saturations below 94%
13	When prescribing oxygen, which must the prescriber complete on the drug chart/prescription?	A. Target saturation range only • B. Signature only • C. Signature, date, and printed name • D. Flow rate only
14	A patient presents with an oxygen alert card. Initial oxygen therapy should be:	A. Withheld until senior review • B. Based on the guidance on the card until blood gas results are available • C. High-flow 15 L/min via reservoir mask regardless • D. Titrated to 94–98% in all cases
15	A complete oxygen prescription, as recommended by the guideline, should include:	A. Target saturation range, signed and dated by the prescriber, with the prescriber's printed name • B. Flow rate and device only • C. Admission oxygen saturation only • D. A fixed FiO₂ and the planned duration of therapy
